# Supplementary material for: Structural layer applicability of semi-flexible material for rutting resistance: A coupled temperature-mechanical approach
Source: PLoS One. 2023 Nov 30;18(11):e0294659. doi: 10.1371/journal.pone.0294659 (PMC10688881; doi:10.1371/journal.pone.0294659)
Supplement: Attachment — Submitted filename: response to comments.docx [file pone.0294659.s006.docx]

**Reviewer #1: The Semi-flexible material (SFM) may be have a performance of rutting resistance. But in summer, asphalt mixture may be in soft state, and cement is a fragile material. So this structure may be cracked because of there material performance.**

**Response:** There are still some distresses in pavement besides rutting, among which cracking is significant. Based on previous studies, the compatibility among components (aggregate, asphalt, and grouting) in SFM is a primary issue. It is an interesting topic, which we will investigate in future works.

**Reviewer #2: The Structural Layer Applicability of Semi-Flexible Material for Rutting Resistance was researched. The coupled temperature-mechanical approach model was instituted by Abaqus. The results are significant. However, the simulation process still needs to be optimized.**

**1. The semi-flexible pavement material combines the rigidity of the cement-based grout. So it exhibits incomplete elasticity. The properties of completely elastic materials are different from those of actual materials. The simulated parameters may not be reasonable.**

**Response:** We assumed the SFM is a semi-flexible material and used a creep model to describe such incomplete elasticity. Further, to validate the creep model, we conducted the accelerated loading test to compare the measured rutting depth and the calculation one in the FE simulation. The results showed little difference between calculated and measured rutting depth. It implies that using the creep model with calibrated parameters can accurately predict the rutting depth of actual roads under temperature load coupling.

**2. In Table 11, the creep parameters of SFM are missing. The creep parameters of SFM are suggested to be investigated by experiments. SFM should also have creep parameters.**

**Response:** We added the creep parameters of SFM in Table 11.

**3. Same mistakes: a) The line number is missing b) The acronym SFP in Table 11 is not defined.**

**Response:** We added the line number. We also checked the whole article and changed the “*SFP*” to “*SFM*” .

**Editor’s comments：**

**1. We note that Figures 1 and 6 in your submission contain copyrighted images. All PLOS content is published under the Creative Commons Attribution License (CC BY 4.0), which means that the manuscript, images, and Supporting Information files will be freely available online, and any third party is permitted to access, download, copy, distribute, and use these materials in any way, even commercially, with proper attribution. For more information, see our copyright guidelines: http://journals.plos.org/plosone/s/licenses-and-copyright.**

**We require you to either (1) present written permission from the copyright holder to publish these figures specifically under the CC BY 4.0 license, or (2) remove the figures from your submission.**

**Response：**Thank you for kind remind. We have changed the Figures 1 and 6 to avoid copyright issues.

**2. It appears that your ORCiD iD has not been validated in your Editorial Manager account and we are unable to proceed until that step is complete.**

**Response:** I have updated the ORCiD to my account.

**3. Thank you for updating your data availability statement. You note that your data are available within the Supporting Information files, but no such files have been included with your submission. At this time we ask that you please upload your minimal data set as a Supporting Information file, or to a public repository such as Figshare or Dryad.**

**Please also ensure that when you upload your file you include separate captions for your supplementary files at the end of your manuscript.**

**As soon as you confirm the location of the data underlying your findings, we will be able to proceed with the review of your submission.**

**Response:** We have uploaded Supporting Information files this time.

**4. If possible, please upload a file showing your changes either highlighted or using track changes. This should be uploaded as a Revised Manuscript w/tracked changes, file type. Please follow this link for more information: http://blogs.PLOS.org/everyone/2011/05/10/how-to-submit-your-revised-manuscript/**

**Response:** We have upload the file named “*Revised Manuscript w/tracked changes*”.

**5. Please note that funding information should not appear in any section or other areas of your manuscript. We will only publish funding information present in the Funding Statement section of the online submission form. Please remove any funding-related text from the manuscript.**

**Response:** We have removed the funding-related text from the manuscript.
