# Supplementary material for: Structural layer applicability of semi-flexible material for rutting resistance: A coupled temperature-mechanical approach
Source: PLoS One. 2023 Nov 30;18(11):e0294659. doi: 10.1371/journal.pone.0294659 (PMC10688881; doi:10.1371/journal.pone.0294659)
Supplement: Attachment — Submitted filename: response to comments.docx [file pone.0294659.s007.docx]

Dear Reviewers:

Thank you for your letter and the reviewers' comments concerning our manuscript. Those comments are all valuable and helpful for revising and improving our paper and the critical guiding significance of our research. The responses to comments are as follows.

**Reviewer #2: The paper has undergone necessary rectifications. However, the following aspects require further discussion:**

**1. In relation to parameter selection, the thermal parameters and Poisson's ratio for the SFM material were aligned with those of conventional composite materials. It is crucial to elaborate on the source of these parameters—whether they were determined through empirical measurements or drawn from references. It is important to note the substantial influence of these parameters on the simulation outcomes.**

**Response:** We detailed the parameter selection in lines 205-206 and 219-223, including the thermal parameters and Poisson's ratio for the SFM. These parameters were drawn from previous works, and the relative references were also cited.

**2. Furthermore, a need for reconfirmation arises concerning the values of dynamic elastic moduli presented in Table 11. The provided data values do not align with the dynamic modulus values; instead, they bear a closer resemblance to static rebound moduli. Additionally, the extent of variation in the dynamic modulus of the SFM material in response to temperature would not exhibit such negligible values. Thus, a thorough reevaluation of the data's accuracy is of utmost importance.**

**Response:** We checked the data in Table 11 and modified the dynamic modulus values. Also, the results were recalculated using the improved parameters. Relevant content based on the simulation results were updated in Abstract, Results and Discussion, and Conclusion (line 306-309, line 322-329, line 339-345, line 349-351, line 366-367, line 371-372, line 374-377, and line 400-408).
